# Activation of the TLR/MyD88/NF-κB Signal Pathway Contributes to Changes in IL-4 and IL-12 Production in Piglet Lymphocytes Infected with Porcine Circovirus Type 2 *In Vitro*

**DOI:** 10.1371/journal.pone.0097653

**Published:** 2014-05-19

**Authors:** Dianning Duan, Shuxia Zhang, Xiaolin Li, Hua Guo, Mengmeng Chen, Yaqun Zhang, Junyuan Han, Yingjun Lv

**Affiliations:** College of Veterinary Medicine, Nanjing Agricultural University, Nanjing, China; Virginia Polytechnic Institute and State University, United States of America

## Abstract

Porcine circovirus type 2 (PCV2) causes immunosuppression in pigs. One causative factor is an imbalance in cytokine levels in the blood and lymphoid tissues. Many studies have reported changes in cytokine production, but the regulatory mechanisms involved have not yet been elucidated. In this study, we investigated alteration and regulation of IL-4 and IL-12 production in lymphocytes following incubation with PCV2 in vitro. The levels of IL-4 decreased and levels of IL-12 increased in lymphocyte supernatants, and the DNA-binding activity of NF-κB and the expression of p65 in the nucleus and p-IκB in the cytoplasm of lymphocytes increased after incubation with PCV2. However, these effects were reversed when lymphocytes were coincubated with PCV2 and the NF-κB inhibitor BAY11-7082. In addition, the expression of MyD88 protein increased and the expression of mRNA for the toll-like receptors (TLRs) TLR2, TLR3, TLR4 and TLR9 was upregulated when lymphocytes were incubated with PCV2. However, no change was seen in TLR7 and TLR8 mRNA expression. In conclusion, this study showed that PCV2 induced a decrease in IL-4 and an increase in IL-12 production in lymphocytes, and these changes were regulated by the TLR-MyD88-NF-κB signal pathway.

## Introduction

Porcine circovirus type 2 (PCV2) is a nonenveloped, single-stranded DNA virus with an unsegmented circular genome. It is the primary causative agent of postweaning multisystemic wasting syndrome (PMWS), which is characterized by generalized lymphadenopathy and tan-mottled, non-collapsed lungs [1]. PCV2 mainly affects weaning piglets, typically at 3–15 weeks, and has a morbidity rate of 5–15% [2]–[3]. In addition, PCV2 can cause immunosuppression in pigs, leading to secondary infection or coinfection with other pathogens, such as *Mycoplasma hyopneumoniae* and porcine reproductive and respiratory syndrome virus (PRRSV) [4]–[5].

Immunosuppression caused by PCV2 is mainly associated with lymphopenia and altered patterns of cytokine expression in blood and lymphoid tissues. The numbers of T and B lymphocytes and CD8^+^ and IgM^+^ cells have been reported to be markedly decreased in the blood, and lymphocyte depletion and histiocytic infiltration are found in lymphoid tissues of PMWS-affected pigs [1], [6]. Lymphocyte depletion is associated with apoptosis and a reduction in lymphocyte proliferation, although the mechanisms are still unknown [7]–[10]. Altered patterns of cytokine expression are also found in piglets infected with PCV2. In pigs naturally suffering from PMWS, IL-10 mRNA in the thymus and IFN-γ mRNA in the tonsils were overexpressed, while expression of IFN-γ, IL-10, IL-12p40, IL-4 and IL-2 mRNA was reduced in other lymphoid tissues [6]. In peripheral blood mononuclear cells harvested from pigs infected with PCV2, expression of IL-2, IL-6, IL-10, IL-12, TNF-γ and TNF-α mRNA was significantly increased and IL-4 mRNA expression slightly decreased compared with healthy pigs [11]. Although cytokine production has been shown to be disordered in pigs infected with PCV2, the underlying regulatory mechanisms remain largely unknown.

IL-4 and IL-12 belong to the Th2-type and Th1-type cytokine families and are associated with humoral and cellular immune responses, respectively. Altered patterns of IL-4 and IL-12 expression can reflect the immune status. Previous studies have suggested that monocyte and macrophage lineage cells and lymphocytes are the main garget cells for PCV2 [12]–[13]. However, lymphocytes are the primary sites for PCV2 replication, while monocyte and macrophage cells are only the reservoir for PCV2 [13]–[14]. In addition, there are more lymphocytes in immune organs compared with monocyte and macrophage cells. Therefore it is necessary to investigate the altered cytokines induced by lymphocytes. In this study, changes in IL-4 and IL-12 expression and the regulatory mechanisms involved were investigated in piglet lymphocytes after infection with PCV2. PCV2 induced a decrease in the level of IL-4, an increase in the level of IL-12, activation of the DNA-binding activity of NF-κB, high expression of p65 in the nucleus and p-IκB and MyD88 in the cytoplasm, and upregulation of toll-like receptor (TLR)2, TLR3, TLR4 and TLR9 mRNA. NF-κB inhibitor abolished the changes in IL-4 and IL-12 expression, DNA-binding activity of NF-κB, and expression of p65 and p-IκB. These data suggest that IL-4 and IL-12 are regulated by the TLR/MyD88/NF-κB signal pathway.

## Materials and Methods

### Virus

The virus isolate PCV2-SH was provided by the Key Laboratory of Animal Disease Diagnosis and Immunology at Nanjing Agricultural University. PCV2-SH has previously been shown to induce PMWS [15]–[16]. The PCV2-SH stock titers were 5×10^5^ TCID_50_ (50% tissue culture infective dose)/mL as determined by titration on PK-15 cells using an immunofluorescence assay.

### Lymphocyte Isolation

All experimental procedures were approved by the Institution Animal Care and Use Committee of Nanjing Agricultural University. Five 35-day-old conventionally weaned piglets were obtained from a pig farm that was negative for PCV2 and PRRSV infections. The pigs were confirmed to be free of PCV2 and PRRSV infections with commercial Enzyme-Linked ImmunoSorbent Assay (ELISA) kits for PCV2 antibody (INGEZIM CIRCOVIRUS IgG/IgM Kit, Ingenasa, Spain) and PRRSV antibody (IDEXX, Westbrook, Maine, USA), and with PCR or RT-PCR for viral nucleic detection. The pigs were euthanized by jugular injection with 10 mg/kg bodyweight of 3% sodium pentobarbital (Sinopharm Chemical Reagent Co. Ltd., Shanghai, China). Lymphocytes were isolated from spleen as previously described [7], [9]. Briefly, spleen tissues were dissociated by forcing through a 70 µm nylon cell strainer using a syringe plunger. Cells were collected from the lymphocyte layer by centrifugation at 2,000 rpm for 20 min at 4°C on lymphocyte separation medium (Sigma-Aldrich, St. Louis, MO, USA). Following removal of residual erythrocytes by ACK lysing buffer and wash with PBS, cells were suspended in RPMI 1640 medium in 250 mL Teflon flasks (Costar, Washington, USA) at a cell density of 5×10^6^/mL with 10% fetal bovine serum, 100 IU/mL penicillin and 100 IU/mL streptomycin. After incubation for 2 h at 37°C in 5% CO_2_, the non-adherent cells were collected and designated as spleen lymphocytes. Prior to use, the cells were confirmed as negative for PCV1, PCV2, PRRSV by PCR or RT-PCR.

### Experimental Design

Lymphocytes were divided into four groups. Group 1 was inoculated with PCV2 virus at 1×10^5^ TCID_50_, Group 2 was coincubated with NF-κB inhibitor (BAY11-7082; obtained from Beyotime Institute of Biotechnology, Jiangsu, China) and PCV2 virus at 1×10^5^ TCID_50_, Group 3 was incubated with BAY11-7082 only, and Group 4 was used as negative control. Lymphocytes from each group were collected at 6, 12, 24 and 48 h for further experiments.

### Indirect Immunofluorescence Assay

Lymphocytes were fixed for 20 min in 4% paraformaldehyde at 4°C and washed with PBS (10 mM, pH7.4). After fixation, lymphocytes were incubated for 1 h with polyclonal anti-PCV2 antibody at 37°C and washed with PBS. Lymphocytes were then incubated with FITC-conjugated goat anti-swine IgG antibody (Sigma-Aldrich), washed again and stained with 5 µg/ml DAPI (Sigma-Aldrich) for 10 min for visualization of nuclei. Finally, cells were washed and examined by fluorescence microscopy (Zeiss, Jena, Germany).

### Flow Cytometry

Flow cytometry was performed to quantitate the numbers of lymphocytes infected with PCV2, and the infection rate was calculated as the ratio of the number of infected cells to the total number of lymphocytes. Briefly, lymphocytes were fixed for 20 min at 4°C with 4% paraformaldehyde and washed with PBS. The cells were then incubated for 20 min with polyclonal anti-PCV2 antibody. After centrifuging and washing, lymphocytes were stained with FITC-conjugated goat anti-swine antibody IgG (Sigma) for 1 h in a dark environment. Stained cells were analyzed using flow cytometer (BD, San Jose, CA, USA).

### IL-4 and IL-12 Assay

The levels of IL-12 and IL-4 in culture supernatants were measured by ELISA using a porcine IL-12p40 ELISA kit (R&D, Minneapolis, MN, USA) and porcine IL-4 ELISA kit (Alpco, Salem, NH, USA), respectively, according to the manufacturer’s instructions.

### Electrophoretic Mobility Shift Assay (EMSA)

Nuclear and cytoplasmic extracts were prepared from lymphocytes using a NE-PER Nuclear and Cytoplasmic Extraction Reagents Kit (Pierce, Rockford, IL, USA) according to the manufacturer’s instructions. Protein concentration was measured using the BCA Protein Assay Kit (Pierce). EMSA was used to detect the DNA-binding activity of NF-κB as we have previously described [16]. Briefly, reaction mixtures (10 µL) containing 15 µg nuclear extract were incubated in reaction buffer for 20 min at room temperature with 10 fmol of biotin-labeled double-stranded oligonucleotide probes (NF-κB p65 oligonucleotides: 5′-CAT CGG AAA TTT CCG GAA ATT TCC GGA AAT TTC CGG C-3′ and 5′-GCC GGA AAT TTC TGG AAA TTT CCG GAA ATT TCC ATG-3′). Samples were then run on a 5% non-denaturing polyacrylamide gel and transferred to a Biodyne B Nylon membrane (Pierce) in 0.5× tris-borate-EDTA buffer (TBE buffer; pH 8.3). The biotin-labeled NF-κB p65 probe was detected using an ImageQuant LAS 4000 biomolecular imager (GE Healthcare Life Sciences, Pittsburgh, PA, USA).

### Western Blotting to Detect NF-κB p65, p-IκB and MyD88

The quantities of p65 in the nuclear protein and p-IκB and MyD88 in the cytoplasmic protein extracts were measured by Western blotting. Protein samples were separated by 12% sodium dodecylsulfate-polyacrylamide gel electrophoresis. Samples were transferred to nitrocellulose membranes (Takara Bio Inc., Otsu, Shiga, Japan), which were then blocked for 2 h in 5% nonfat dry milk suspended in 0.1% Tween-20 Tris-buffered saline (TTBS, pH 7.4). Membranes were incubated overnight at 4°C with monoclonal antibodies against NF-κB p65 (Enzo Life Sciences, Farmingdate, NY, USA), p-IκB (Enzo Life Sciences), MyD88 (Abcam, Hong Kong), histone H3 (Santa Cruz Biotechnology, Dallas, Texas, USA), glyceraldehyde 3-phosphate dehydrogenase (GAPDH; Abcam, USA) or β–actin (Cell Signaling Technology, Boston, MA, USA). Membranes were washed in TTBS, incubated with horseradish peroxidase-conjugated secondary antibody (Univ-bio, Shanghai, China), and developed using enhanced chemiluminescence reagents. Signals were captured and measured using the ImageQuant LAS 4000 biomolecular imager.

### Quantitative Real-time RT-PCR Detection of TLRs

RNA was extracted from lymphocytes using RNAiso Plus (Takara Bio Inc.) according to the manufacturer’s instructions. The RNA samples were then reverse-transcribed into cDNA using the PrimeScript RT Master Mix Perfect Real-Time Kit (Takara Bio Inc.). The PCR primers were designed using Primer Premier 5.0 software and the primers for β-actin and TLR2, 3, 4, 7, 8 and 9 are shown in Table 1. The PCR reaction system was consisted of 2 µL cDNA, 10 µL of 2× SYBR Premix Ex Taq (DRR041S; Takara Bio Inc.), 0.6 µL of sense primer, 0.6 µL of anti-sense primer and 6.8 µL double-distilled water, following the manufacturer’s protocol. Quantitative PCR was performed using the Bio-Rad IQ5 qPCR thermocycler (Bio-Rad, Berkekey, CA, USA). Two replicates were performed in the experiments.

**Table 1:** Primer sequences used in this study for real-time PCR in pigs.

Gene	Accession number	Primer sequence (5′→3′)	Expected PCR products size
*β-actin*	U07786	Sense: CTCCATCATGAAGTGCGACGT	114
		Anti-sense: GTGATCTCCTTCTGCATCCTGTC	
*TLR2*	NM_213761.1	Sense: TCATCTCCCAAATCTGCGAAT	167
		Anti-sense: GGCTGATGTTCTGAATTGACCTC	
*TLR3*	NM_001097444.1	Sense: GCAAGAACTCACAGGACAGGAA	100
		Anti-sense: GGCGAAAGAGTCGGTAGTCAA	
*TLR4*	NM_001113039.1	Sense: CCGTCATTAGTGCGTCAGTTCT	100
		Anti-sense: TTGCAGCCCACAAAAAGCA	
*TLR7*	NM_001097434.1	Sense: TTGTTCCATGTATGGGCAGA	238
		Anti-sense: TTCCAGGTTGCGTAGCTCTT	
*TLR8*	NM_214187.1	Sense: TTCCCACATCCCAGACTTTC	202
		Anti-sense: TTGCTTTGGTTGATGCTCTG	
*TLR9*	NM_213958.1	Sense: AGCCTCAACCTGTCCTTCAATTACC	128
		Anti-sense: CTGAGCGAGCGGAAGAAGATGC	

### Statistical Analysis

All experiments were repeated at three times with the same splenic lymphocytes from the five pigs. Statistical analysis of experimental data was performed using SPSS v18.0 software. Results are presented as mean ± standard deviation. One-way ANOVA was used to determine the significance of differences between groups. *P* values of 0.05 or 0.01 were taken to be statistically significant, and are labeled with an asterisk (*) or (**) on each graph.

## Results

### Lymphocyte Infection

**Figure 1:**
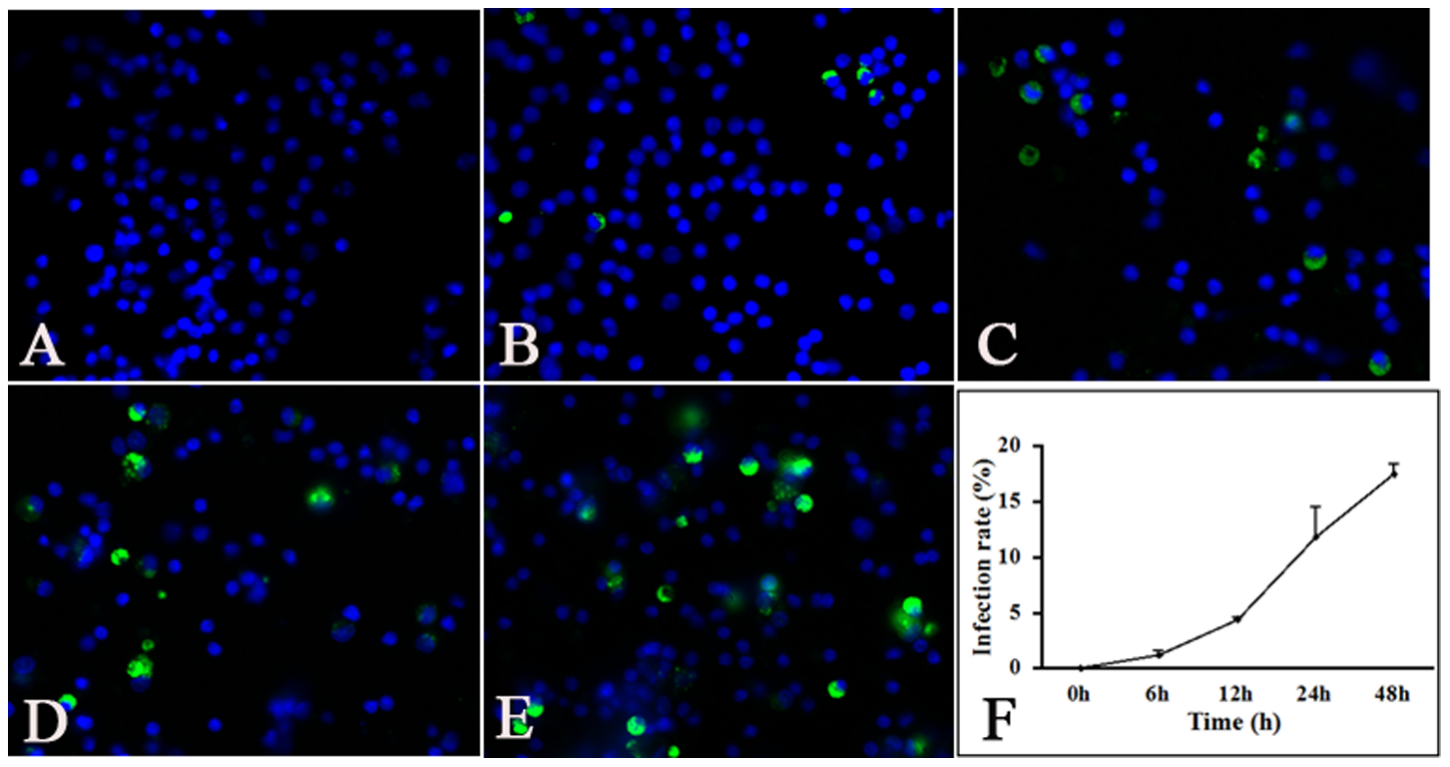
PCV2 infection in piglet lymphocytes. Control lymphocyte cultures (A) and lymphocytes incubated with PCV2 for 6, 12, 24 and 48 h (B–E) were stained with anti-PCV2 antibody (green) by indirect immunofluorescence. DAPI was used to visualize the nuclei (blue). (F) The percentage of infected lymphocytes at each time point was quantified by flow cytometry. The numbers of infected cells increased with incubation time.

To determine whether lymphocytes were infected with PCV2, viral antigens were detected by immunofluorescence. PCV2 antigens were detectable after incubation of lymphocytes with virus for 6, 12, 24 and 48 h, while no PCV2 antigens were found in the control group (Fig. 1A–E). The number of infected lymphocytes increased with incubation time. Flow cytometry was used for quantitative assessment of the numbers of infected cells (Fig. 1F). After 6, 12, 24 and 48 h of incubation with PCV2, the percentage of infected lymphocytes cells was 1.19%, 4.42%, 11.84% and 17.48%, respectively.

### Effect of PCV2 on the Production of IL-4 and IL-12

**Figure 2:**
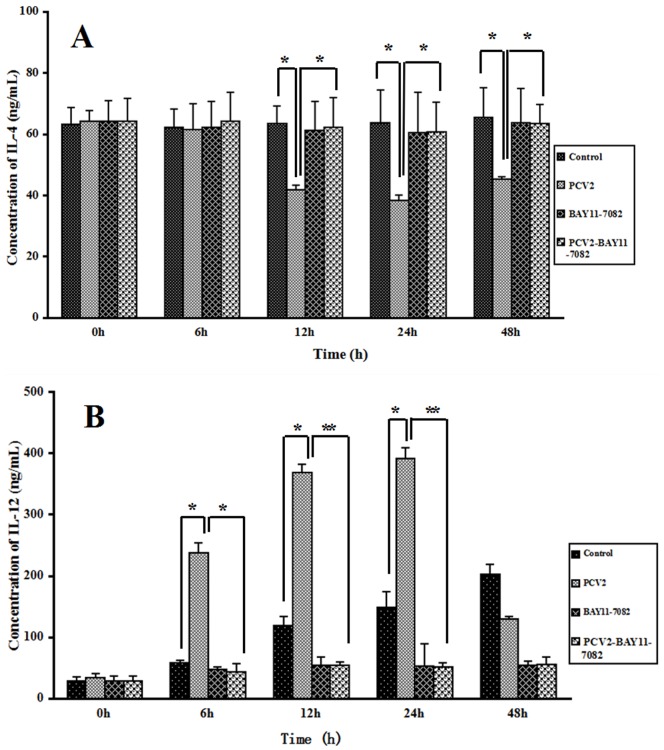
Changes in IL-4 and IL-12 levels after PCV2 infection. Supernatants from control cultures and lymphocytes cultured with PCV2, NF-κB inhibitor BAY11-7082, or a combination of both, were sampled after 6, 12, 24 and 48 h and tested by ELISA to determine the concentration of (A) IL-4 and (B) IL-12. PCV2 induced a decrease in IL-4 and an increase in IL-12 concentration, and these effects were abrogated by BAY11-7082. * *P*<0.05, ** *P*<0.01.

The levels of IL-4 and IL-12 in the culture supernatant were determined by ELISA (Fig. 2). The level of IL-4 in the PCV2 group after 12, 24 and 48 h of incubation was lower than in the control group (*P*<0.05; Fig. 2A), and the level of IL-12 in the PCV2 group after 6, 12 and 24 h of incubation was higher than in the control group (*P*<0.05), suggesting that PCV2 infection led to decreased IL-4 and increased IL-12 secretion in lymphocytes. The levels of IL-4 after 12, 24 and 48 h in the group coincubated with PCV2 and BAY11-7082 were higher than in the PCV2 group, and the levels of IL-12 after 6, 12 and 24 h in the PCV2 and BAY11-7082 coincubation group were lower than in the control group (*P*<0.05) (Fig. 2B). These results demonstrate that effects of PCV2 on the levels of IL-4 and IL-12 were reversed by the NF- κB inhibitor BAY11-7082.

### Effect of PCV2 on the DNA-binding Activity of NF-κB

**Figure 3:**
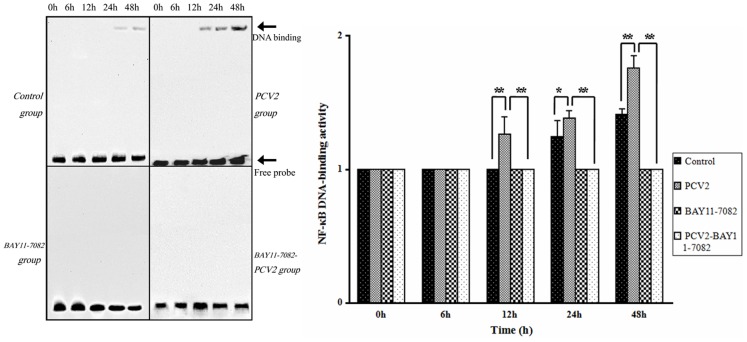
Changes in NF-κB DNA-binding activity after PCV2 infection. An electrophoretic mobility shift assay (EMSA) was used to measure NF-κB DNA-binding activity after 6, 12, 24 and 48 h in controls and lymphocytes incubated with PCV2, NF-κB inhibitor or both. PCV2 increased NF-κB DNA-binding activity, and this effect was abrogated by BAY11-7082. * *P*<0.05, ** *P*<0.01.

To determine whether NF-κB signaling was activated after lymphocytes were infected with PCV2, EMSA was used to detect the ability of NF-κB to bind to DNA (Fig. 3). The binding activity of NF-κB in the BAY11-7082 group didn’t change during the incubation and was same as the binding activity in the control group after 0 h of incubation, showing that BAY11-7082 completely inhibited NF-κB activation. The binding activity of NF-κB in the PCV2 group after 12, 24 and 48 h of incubation was higher than in the control group (*P*<0.05), indicating that NF-κB signaling was activated by PCV2. However, the binding activity of NF-κB in the group coincubated with PCV2 and BAY11-7082 was lower than in the PCV2 group, demonstrating that BAY11-7082 inhibited NF-κB activation caused by PCV2.

### Effect of PCV2 on NF-κB p65 and p-IκB

**Figure 4:**
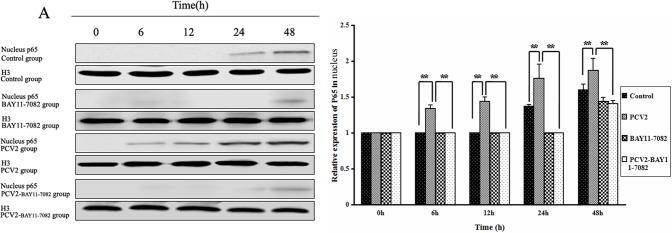
Changes in NF-κB p65 protein expression after PCV2 infection. Western blotting was used to measure expression of NF-κB p65 protein in the nucleus at 6, 12, 24 and 48 h in each group of lymphocytes. Levels of p65 increased after PCV2 infection and this effect was abrogated by the NF-κB inhibitor BAY11-7082. Expression of histone H3 was used as a positive control. ** *P*<0.01.

**Figure 5:**
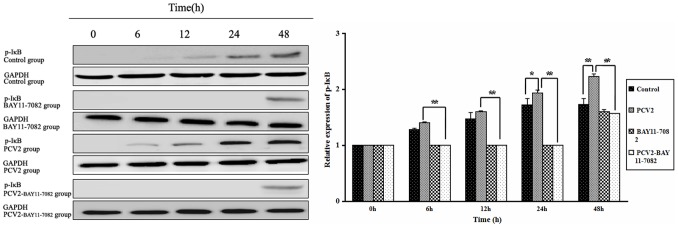
Changes in p-IκBα protein expression after PCV2 infection. Western blotting was used to measure expression of p-IκBα protein in the cytoplasm at 6, 12, 24 and 48 h in each group of lymphocytes. Levels of p-IκBα increased after PCV2 infection relative to controls, and this effect was abrogated by the NF-κB inhibitor BAY11-7082. Expression of GADPH was used as a positive control. * *P*<0.05, ** *P*<0.01.

To further investigate whether NF-κB signaling was activated after lymphocytes were infected with PCV2, levels of p65 and p-IκB proteins were measured by Western blotting (Figs. 4 and 5). The levels of p65 protein in the nucleus in the PCV2 group after 6, 12, 24 and 48 h of incubation were significantly higher than in the control group (*P*<0.01; Fig. 4), and the levels of p-IκB protein in the cytoplasm in the PCV2 group after 24 and 48 h were higher than in the control group (*P*<0.05; Fig. 5), indicating an increase in translocation of NF-κB from the cytoplasm to the nucleus. In lymphocytes coincubated with PCV2 and BAY11-7082 for 6, 12, 24 and 48 h, both the nuclear p65 protein levels (Fig. 4) and the cytoplasmic p-IκB protein levels (Fig. 5) were significantly lower than in the PCV2 group (*P*<0.01), demonstrating that BAY11-7082 inhibits the translocation of NF-κB.

### Effect of PCV2 on MyD88

**Figure 6:**
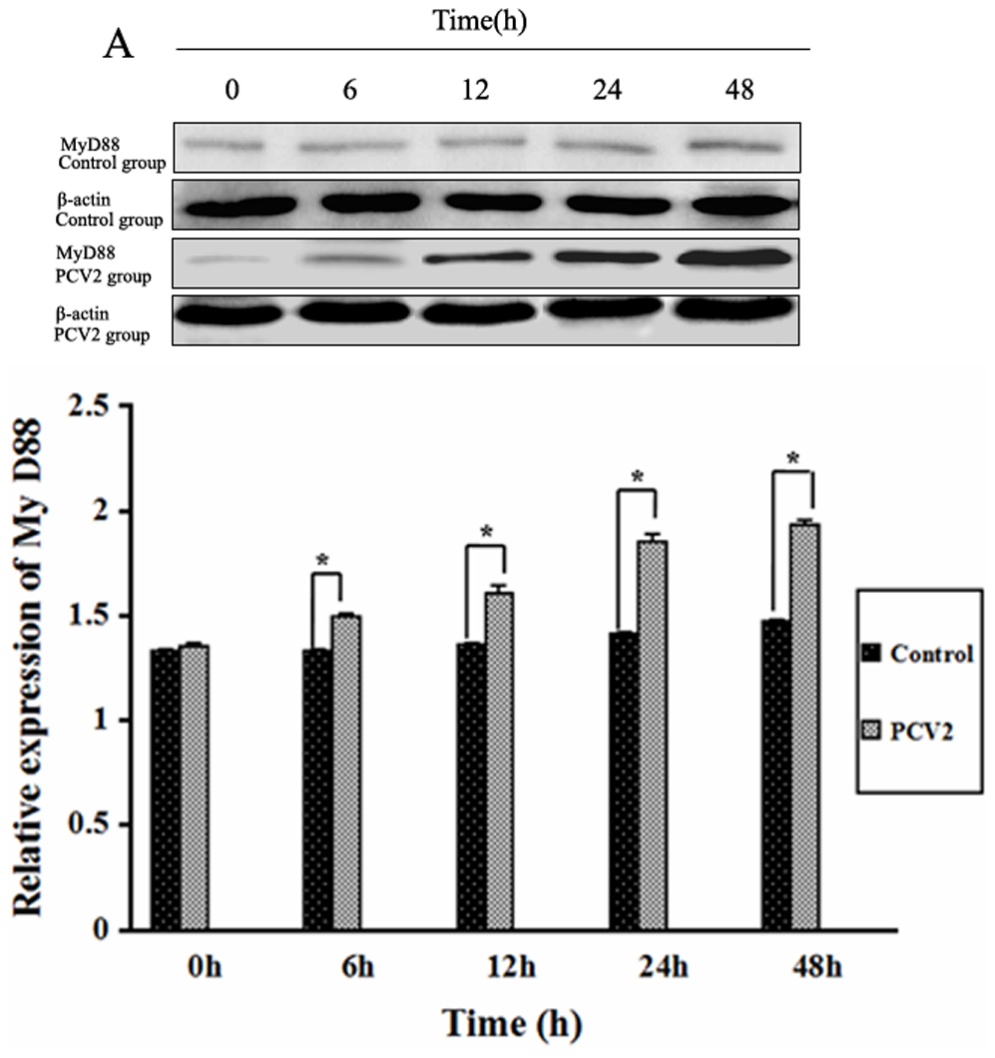
Changes in MyD88 after PCV2 infection. Western blotting was used to measure expression of MyD88 protein after 6, 12, 24 and 48β-actin was used as a positive control. * *P*<0.05.

To determine whether MyD88 was activated after lymphocytes were inoculated with PCV2, levels of MyD88 protein were measured by Western blotting (Fig. 6). The levels of MyD88 protein in the PCV2 group after 6, 12, 24 and 48 h were significantly higher than in the control group (*P*<0.05).

### Effect of PCV2 on TLR mRNA Expression

**Figure 7:**
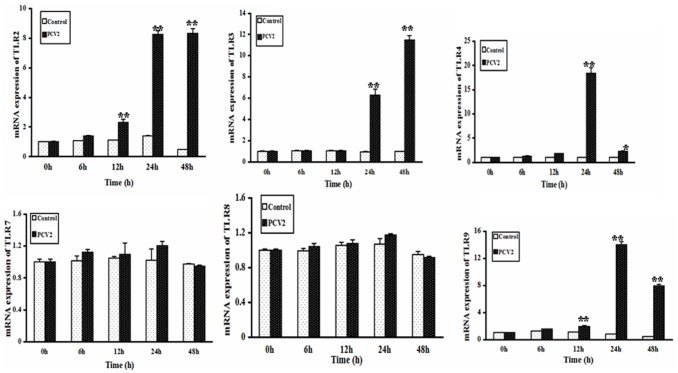
Changes in expression of TLR mRNA after PCV2 infection. Real-time RT-PCR was used to assess the mRNA expression of TLR2, 3, 4, 7, 8 and 9 in lymphocytes incubated with PCV2 and controls after 6, 12, 24 and 48 h. Increased expression of TLR2, TLR3, TLR4 and TLR 9 mRNA was seen in infected cells. There was no significant change in TLR7 and TLR8 mRNA expression. * *P*<0.05, ** *P*<0.01.

To determine whether TLRs were activated after lymphocytes were inoculated with PCV2, TLR mRNA expression was measured by real-time RT-PCR (Fig. 7). The expression of TLR2 and TLR9 mRNA in the PCV2 group was significantly higher than in the control group at 12, 24 and 48 h (*P*<0.01). The expression of TLR3 and TLR4 mRNA in the PCV2 group was significantly higher than in the control group at 24 and 48 h (*P*<0.05). However, there were no statistically significant differences in the expression of TLR7 and TLR8 mRNA between the PCV2 and control groups (*P*>0.05).

## Discussion

PCV2 has been shown to cause immunosuppression in pigs [16]–[17]. One reason for this is the induction of cytokine imbalance in the blood and lymphoid organs [1], [6], [11], [17]. Although many studies have investigated changes in cytokine production in PCV2-infected pigs both in vitro and in vivo, the regulatory mechanisms behind these changes are still unknown. In this study, we chose to investigate the mechanism of regulation of IL-4 and IL-12 expression following infection of lymphocytes from pig spleen with PCV2. IL-4 levels decreased from 12 h and IL-12 levels increased from 6 h after inoculation of lymphocytes with PCV2, demonstrating that PCV2 infection led to an imbalance in cytokine production, in line with previous studies [6], [11].

NF-κB is a nuclear transcription factor that regulates the expression of a large number of genes including those involved in inflammation. NF-κB is sequestered in an inactive form in the cytoplasm via its association with the inhibitory κB (IκB) complex. Following activation, NF-κB is released into the cytoplasm and translocates to the nucleus where it initiates gene transcription, resulting in the production of various cytokines [18]–[19]. Following the infection of lymphocytes with PCV2, this study showed an increase in the expression of p-IκB (from 24 h after inoculation), the expression of p65 in the nucleus (from 6 h), and NF-κB DNA-binding activity (from 12 h) compared to controls, demonstrating that NF-κB signaling was activated in lymphocytes by PCV2 infection, a result consistent with previous reports [16], [20]–[21]. To determine whether the changes in cytokine production were related to NF-κB signal activation, the NF-κB inhibitor BAY11-7082 was used in further experiments. Compared with lymphocytes incubated with PCV2 alone, those incubated with PCV2 and BAY11-7082 showed decreased expression of p-IκB, decreased p65 in the nucleus (from 6 h), and decreased NF-κB DNA-binding activity (from 24 h), demonstrating that BAY11-7082 inhibited the activation of NF-κB. Compared with lymphocytes incubated with PCV2 alone, those incubated with PCV2 and BAY11-7082 showed increased levels of IL-4 (from 12 h to 48 h) and decreased levels of IL-12 (from 6 h to 24 h), indicating that the changes in IL-4 and IL-12 expression were regulated via the NF-κB signal pathway.

TLRs and MyD88 are upstream regulatory factors of NF-κB [22]. The TLR family is an essential component of the array of signaling receptors expressed on the cell surface, and distinguishes pathogen-associated molecular patterns (PAMPs), shared widely by microorganisms, from host molecules. After stimulation with an appropriate ligand, TLRs relay the signal via MyD88, a common signal adaptor molecule shared by all members of the TLR family except TLR3, which relays signals via TRIF or ticam-1, to activate NF- κB [23]–[26]. In this study, the expression of MyD88 protein and TLR2, TLR3, TLR4 and TLR9 mRNA increased after lymphocytes were incubated with PCV2, while expression of TLR7 and TLR8 mRNA was unchanged compared with controls, demonstrating the involvement of TLRs/MyD88 signaling in lymphocyte responses to PCV2 infection. It has been reported previously that activation of TLRs/MyD88 is involved in the production of cytokines in pigs. The levels of TNF-α, IL-1, IL-6, IL-10 and IFN-γ in alveolar macrophages and peripheral blood mononuclear cells increased and the mRNA expression of TLR2, 3, 4, 7 and 8 were upregulated in PRRSV-infected pigs [20], [27]–[29]. MyD88 played an important role in IL-10 production during PRRSV infection, and the induction of IL-10 was dependent on NF-kB activation and p38 MAPK [27]. In this study, the decrease in IL-4 and increase in IL-12 production caused by PCV2 infection may also be regulated by TLRs and MyD88, although further research is necessary to investigate this in more detail.

In this study, PCV2 infection in piglet splenic lymphocytes induced a decrease in IL-4 levels, an increase in IL-12 levels, activation of NF-κB and increased expression of MyD88, TLR2, TLR3, TLR4 and TLR9. Blockage of NF-κB abrogated the effects of PCV2 on cytokine production, resulting in increased IL-4 and decreased IL-12 levels compared with PCV2 alone. These data indicate that the changes in IL-4 and IL-12 production are regulated by the TLRs/MyD88/NF-κB signal pathway.
